# Supplementary material for: Identification and characterization of expression profiles of neuropeptides and their GPCRs in the swimming crab, Portunus trituberculatus
Source: PeerJ. 2021 Sep 15;9:e12179. doi: 10.7717/peerj.12179 (PMC8449533; doi:10.7717/peerj.12179)
Supplement: Supplemental Information 2 [file peerj-09-12179-s002.docx]

Table S2 Primers for tissue distribution of *P.* *trituberculatus* neuropeptide genes used for RT-PCR.

| Neuropeptide genes | Primer sequences（5^’^-3^’^） |
| --- | --- |
| ACP | F-GGGTGCCACAGGGGAAGAG  R-TGACCAGCAAGGGAGGAGAGA |
| ALP | F-CCACCGCCCTAACGACTG  R-CCCCCACTGCTGGAAGATAC |
| AST-A | F-CTCCCCCTGTCCGTGGT  R-GGTCGTACTGAAGGTTATAGTTCCC |
| AST-B | F-ACAGCGAAGACATTGACGACG  R-TTCCCCAAAGCGCCCAC |
| AST-C | F-GTGACCTACTGAAGGGGGAGA  R-CCAGAGAGAGGGGCAAACAT |
| AST-CCC | F-GAGGAGGATGGCTCGCTG  R-GAGGAGGATGGCTCGCTG |
| CCAP | F-AGCAAGGTTATGGGAGCAA  R-ATACTGTGGCTGAATAAGAGACC |
| CHH1 | F-ATCCGTATGCCAAGTGAGCC  R-TGAGGGACGCCAGCAGTCT |
| CHH2 | F-AGTCTATCAAATCCGTATGCCAA  R-CAGTCTGCCCATTCTTCCA |
| MIH | F-GTATTGCTGGCTGTCCTTTG  R-GCAGATCCATTCTACCTTTTTGT |
| CFSH | F-CCATACTTTACAGAACAAGAGAGCA  R-TGAACAGTTGACACCACGATG |
| Corazonin | F-CAGTTGTGGTGCTCGTTGCC  R- GCTCAGCGGACCTTTTTCG |
| CNMamide | F-GCGGAGGCAAGCAATAAAAAC  R-CAGGTCGTCCCAGGGAAGG |
| DH31 | F-TCGGTCTGTGTCTGGTGGTT  R-GCATAAGATACTCCCTCATCTTCTCA |
| Elevenin | F-CACACCCATCTCAGTCATTGC  R-GGTTCGCTCCACCTACACG |
| GPA2 | F-TGTCTGCTCGCCTACTCCTC  R-ATGTCAAACTCAACACACTCTGG |
| GPB5 | F-TCTGCCTCCTTCCTCCAGC  R-GCACATCCTCCCGTTATCGT |
| GSEFLamide | F-ACAGAATGACGCCGGAGAC  R-TTGTTGAGGAGGAGGGAGAAG |
| Kinin | F-GGTGGGTTTGATGATGTGCTAC  R-ATGCTTTCCGCTTTGTGTC |
| Myosuppressin | F-CTATTCCTCCTCCCATCTGC  R-AGTCCTCCATTGCTCGTGA |
| Neuroparsin1 | F-GGCGAGACTTGTGGAGGGTA  R-GGAGGCGTCAAAGGGAGAA |
| Neuroparsin2 | F-TGATTGTGATGATGGTGGGC  R-TGGGTGCAACGCGAGAC |
| Neuroparsin3 | F-ATCGTCTCACTTCTTTTCTTTCC  R-GTGTTGCCACAGCGGTTC |
| NPF1 | F-GCTGGCTGGCACACTGAG  R-GTAGGCATTGAGGGCGTCT |
| NPF2 | F-GCGATTCGGGAAGCGTAGC  R-CGCCTCCTTGCCAGTGTCT |
| sNPF | F-GCTGGCAGGTGTCTCAGAGGT  R-TCGTGGTGGTCGAGGATGG |
| PDH1 | F-ACCAAGGGGCAGGAGCT  R-TTGCGTTTGTGAGGGAGACC |
| PDH2 | F-CGCCGTGGTCGTAGCCG  R-CGCCACCATCTCACGCTCTT |
| Ryamide | F-AGAGCGAGGAAGGCGAAGT  R-CGGATGCGGATTTTTTGAGG |
| RPCH | F-GTGGCGTGGGAGAGGCA  R-GACAGCTGAGACAGGGATAGGG |
| Tachykinin | F-AAGAGTGAAGAAGAGGAAGAGGAGG  R-CACGAGACTGTCAAATTCGGAGT |
| Trissin | F-ACGAAATGCTGGAGCTGGAGG  R-GGGGGTGGCTGGTGGAAA |
| β-actin | F-CGAAACCTTCAACACTCCCG  R-GGATAGCGTGAGGAAGGGCATA |

Table S3 Primers for tissue distribution of *P.* *trituberculatus* neuropeptide GPCR genes used for RT-PCR.

| Neuropeptide GPCR genes | Primer sequences（5^’^-3^’^） |
| --- | --- |
| Pt-GPCR-A1 | F-TTCGCCCGCAACAAGCA  R-GAGCCTGACCGTCCAGCAAC |
| Pt-GPCR-A2 | F-CCTTCGGGAGCATCTGGT  R-CGTTGCGTTCTGTGCGTAT |
| Pt-GPCR-A4 | F-TACGACGCTATCACCCATCC  R- CGCCGTCCACTGATGTCTC |
| Pt-GPCR-A7 | F-CGCAACTACACTAACGGCAAGA  R-CGCCACGACTGTCCACAAAA |
| Pt-GPCR-A8 | F- ACCCGATCAAAGGCAAGG  R-CAAGATGTGACTGTCCCGAAT |
| Pt-GPCR-A9 | F-CAAGCACCTCAGAAACATCCG  R-AACAGCGTGGTCACCCAAAA |
| Pt-GPCR-A11 | F-GCTGGGTGTTCGCCCTTAC  R-CCACGGATGGCGCAGTAG |
| Pt-GPCR-A12 | F-AGCCCGTCTTGTTTACCTTGTG  R-TGCTGAACTTGCGCTGTCTTAC |
| Pt-GPCR-A13 | F-GAGGAATGAGTGGCGGATAAC  R-GGACGTGCAGACCTGGGTA |
| Pt-GPCR-A14 | F-GGAGCTGAATCTTGCGAGGA  R-GGACTTGGAGTGCTTGTAGGGA |
| Pt-GPCR-A16 | F-CTCCCTTCAGAGGAATGTCTCG  R-CTGTGGTTGCTGTGGCTCAT |
| Pt-GPCR-A18 | F-GCTGACTTCAACTACGCAGGATT  R-GTTGAGCCAGCCGTACAGAA |
| Pt-GPCR-A19 | F-TGTTGGCTGGACTTTGGGTGA  R-GGCGCAAGGCAGAAGGTGAA |
| Pt-GPCR-A21 | F-CGGCGAAGCCTCCGTTAGAT  R-TGGGACACGAAGGTGGTCAT |
| Pt-GPCR-A23 | F-TGAGTCGGTCAACCAAGGAG  R-GCGTGGGTGACAAGAATACAG |
| Pt-GPCR-A24 | F-CACGCCTGCTACAACCCTC  R-CTCTGCAATCCCGCTGACT |
| Pt-GPCR-A25 | F-AACCCGTTACAGGAGCAGC  R-CAGCCTCAGTAGAGCAATGACTA |
| Pt-GPCR-A26 | F-CGGCGAAGCAGCGACTA  R-CGTATCCCATGAACCACCC |
| Pt-GPCR-A27 | F-TGAGGACGAGGACGAAGAGGA  R-TGGGCTGGCGGGAAATAAAG |
| Pt-GPCR-A28 | F-ATCATCCCGTGTCTGCTCC  R-GCTTGCGTTCTGCTTCCAT |
| Pt-GPCR-A29 | F-TGCTGGAGGAGGTGCTTACTT  R-CGTCTGTTTCGCTGGAACTTATTG |
| Pt-GPCR-A32 | F-AGAGGGTGGTGAAGATGGTGA  R-GTTGAGGGCGGAGTTGGA |
| Pt-GPCR-A33 | F-CTTACGAGGCGGACAGCAAC  R-GGTGAGTTCCCAGGCGACA |
| Pt-GPCR-A34 | F-AGGGTCACGTACAATTCCTGG  R-TTCGCTTCTGAGCCTCTTTG |
| Pt-GPCR-A36 | F-CGCTTCGGCTTCGTCTACTG  R-ACGATGAGGATGGTGGCTTTC |
| Pt-GPCR-A37 | F-ACAGGCGGCTCCTCAACC  R-CCGAAGGCAAGGGTCCAAGT |
| Pt-GPCR-A39 | F-TTGCTACCCGCTATCAAACTG  R-CGGAATGAAAGACGCCCAAC |
| Pt-GPCR-B2 | F-GGCACTTCGGTCTCCTTCAC  R-GAGTAGTCTGTTATCTCCGCTCC |
| Pt-GPCR-B4 | F-GCGGACTTAGGGTGGGAG  R-TCAGGGAGTGGAATCTGTGC |
| Pt-GPCR-B5 | F-GTTCGACGGCGTGTCCTG  R-CGGCGGGTTGGTAATGG |
| Pt-GPCR-B6 | F-CCTCAACTTTCATTCCGCCTTGT  R-CGCCGCACTCCTTCCTCCT |
| Pt-GPCR-B7 | F-CCAGCAGGCGAAGAAGG  R-GGTGAGGAAAGTGGTAACGAAAGA |
| Pt-GPCR-B8 | F-TGCTTCACCAAACCCATACAGG  R-GCGAAAGACTCAGGCCGATTA |
| β-actin | F-CGAAACCTTCAACACTCCCG  R-GGATAGCGTGAGGAAGGGCATA |
