# Supplementary material for: Identification and characterization of expression profiles of neuropeptides and their GPCRs in the swimming crab, Portunus trituberculatus
Source: PeerJ. 2021 Sep 15;9:e12179. doi: 10.7717/peerj.12179 (PMC8449533; doi:10.7717/peerj.12179)
Supplement: Supplemental Information 4 [file peerj-09-12179-s004.doc]

1、*Portunus trituberculatus* prepro-adipokinetic hormone-corazonin-like peptide(full-length protein)

MASWMLAALVVSCVLVGSVTPQITFSRSWVPQGKRSSPTGDIPEPLDPCRDARAATLSSLAGHLLDMMNDVAAADHRPLPDDGTTALRLRNAMMDRRRRVA

2、*Portunus trituberculatus* prepro agatoxin-like peptide

MGSKTAVMILALSLLVSVVLAQPLLEEGREPDGLQQAEVDYTADLLDHLLGRAQNREDTAAMSRDKKWRSCIRRGGACDHRPNDCCYNSSCRCNLWGTNCRCQRMGIFQQWGK

3、*Portunus trituberculatus* prepro-allatostatin A

MRRGSLLPLSVVVVVVVVVGLPPPATAQLGNYNLQYDPNTLLQILQQYEAAAAEAEAAAVAAAEVEAAAVEELEDEDSYSYGKRQIPNHYSFGLGKRTPNAYSFGLGKRGGVYSFGLGKKSGNYNFGLGKRSVRDVASEDQEKQALEEEDKDEVKRSKRDVSEEKEEEEDKRTAYDTYSKRPRAYSFGLGKRKQEDMAFSKRPRDYAFGLGKREYTDLEKRPRSYSFGLGKREDDQELEKRQRPYSFGLGKREDDSEFDKRQGPYSFGLGKRGYDDEEEEDKTFYAFGLGKRPRTYSFGLGKRADDLEEEEEEEEEEPLDLDELEDLAKRASGIQNYDFGLGKRAGPYSFGLGKRDGPYSFGLG

KRDGPYSFGLGKRDGPYSFGLGKRGGLYEFGLGKRGSGQYAFGLGKKAGQYSFGLGKRAQGDSSDSYTLGRRSGSYSFGLGKRAGPYSFGLGKRAGPYEFGLGKRAGPYSFGLGKRAGPYSFGLGKRAGPYSFGLGKRAGPYEFGLGKRAGPYSFGLGKRAGPYSFGLGKRAGPYEFGLGKRAGPYSFGLGKRAGPYEFGLGKRAGPYEFGLGKRPSDGMYAFGLGKRGGPYSFGLGKRGGPYSFGLGKRGGPYSFGLGKRDVDEEEQDKMVLENQASSGDSSSSSSPSSSSSSS

4、*Portunus trituberculatus* prepro-allatostatin B(full-length protein)

MQLATLTATLLTLVAAAAAQDEGSGLAVAQAKRAGWSSMRGAWGKRDDSSDQGLQVSEDKRNNNWRKFQGSWGKRGEEIQDAEDKRGNWNKFQGSWGKRADDITEEAALQAAEEKRGGWNKFQGSWGKRGDEVASEDDLQDAEDKRTSWGKFQGSWGKRQDDLIQLQDLEDKRNNWSKFQGSWGKRAGWSSLQGAWGKRAWSNLQGAWGKRSPNDSEDIDDEALEEEELQVSPEALARMVAASPVKRGWALWGKRPDYPAVSPRSTNWSSLRGTWGKRSGDWSSLRGAWGKRVPNDWAHFRGSWGKRSPDTLIA

5、*Portunus trituberculatus* prepro-allatostatin C(full-length protein)

MMPCLGHLVVALALVLTLSHALPAKEVPEAQKEVSTAHEGGRLQKRAASPSSDTNQEELAVLKDLILSRLAAELDDTYQDLPSFKRDLLKGEIDGEEEDEGNEEGRREDGKKKRMFAPLSGLPGNLRTIKRQIRYHQCYFNPISCFRRK

6、*Portunus trituberculatus* prepro- allatostatin CCC(full-length protein)

MSPRLSTVLILAVVVLAALGTTSAKPLGEQDPSAGGPPAFTAREAQVYEPYGNNLEEDGSLDAALINYLFAKQLVQRLRSPSEVSRESQRKRSYWKQCAFNAVSCFGKRK

7、*Portunus trituberculatus* prepro- bursiconα(full-length protein)

MSSNLAWAVVGATVTVLVVIGVDVARADECSLRPVIHILSYPGCTSKPIPSFACQGRCTSYVQVSGSKLWQTERSCMCCQESGEREAAITLNCPKPRPGEPKEKKVLTRAPIDCMCRPCTDVEEGTVLAQEIANFIQDSPMDSVPFLK

8、*Portunus trituberculatus* prepro- bursiconβ(full-length protein)

MWCGRGLLAAAVVVVVAVLLPNTVHCRTYGIECETLPSTIHISKEEYDDTGRLVRVCEEDVAVNKCEGACVSKVQPSVNTPSGFLKDCRCCREVHLRARDITLTHCYDGDGARLTGAKATQQVKLREPADCQCFKCGDSTR

9、*Portunus trituberculatus* prepro-crustacean cardioactive peptide (full-length protein)

MYFTSLSGRAGLIIAGTILLMAFLIADTEGGTVAKRDIDSLLDGKMKRPFCNAFTGCGKKRSDPELEGLASGSELDDITKHVLAEARLWEQLQNKMEAMRILASRMDSRPVFRRKRSLIQPQYNHANSAATLKHKGVVEKQ

10、*Portunus trituberculatus* prepro-crustacean hyperglycemic hormone I (full-length protein)

MQSIKSVCQVSLVAACIIFTLPWTQARSAEGFGRMGRLLASLKADSLTPMQGYGTETGHPLEKRQIYDSSCKGVYDRAIFSELEHVCDDCYNLYRTSRVASGCRSNCYSNMVIRQCMEDLLLMDNFEEYARKIQMVGKK

11、*Portunus trituberculatus* prepro-crustacean hyperglycemic hormone Ⅱ (full-length protein)

MQSIKSVCQVSLVAACIIFTLPWTQARSAEGFGRMGRLLASLKADSLTPMQGYGTETGHPLEKRQIYDSSCKGVYDRAIFSELEHVCDDCYNLYRTSRVASGCRENCFENDLFEECVFELMLPDEMFLIRDAIRG

12、*Portunus trituberculatus* pre-molt-inhibiting hormone I(full-length protein)

MVSRAHSRFSCQRTTLLAVVLLAVLWSSSLQQAAARVINDDCPNLMGNRD

LYKKVEWICDDCANIYRSTGMASLCRKDCFFNEDFLWCVRATERSEDMMQ

LKQWVRILGAGRI

13、*Portunus trituberculatus* prepro-CCHamide I (full-length protein)

MQTWARISTLLLLVLPFLLVLTPAAEAHGSVKVGCLNYGHSCLGAHGKRGSWPPAAPRTQTAAALLAPFLQGLAAPRNAVRSWQDKVMSQRRQQEKEQQQPQQQQQHHQDDFPVMYEDSPLPRIPAARHNALPANQDTSLDDDDLVYYGTYEEDYGDHRAKRSVTEKTEKQKTTPTKVSMSCLLFSRRC

14、Portunus trituberculatus prepro-CCHamide Ⅱ (full-length protein)

MARVALSVVLCLTVASSVLSVAEGKCSQFGHSCFGAHGKRGGEQYGSLDPADLYPPSNQLAEVDEARYALEEAPVTNPRNMVKVRDLMDLLSHLLRQRPAPLQQQQQRSPLPPIPAQLGPNDAYLH

15、*Portunus trituberculatus* prepro-crustacean female sex hormone（C-terminal partial protein）

+QNGASLRLTSFIIFLLLECSFAYNPVYPLRVQHFLQYLQQERQQLVTSLTSRSPKRSSIIGHMNSILYRTREQVMEGMMDTYILVPRAIISSTAKLHRGVNCSEYREVSKVYGNGFEANYNLRPTWLHRSKTVSTCPTQYIERQIQGPLPIQPVTILEAKCVCEGSQCSQDGSICVAVKYRLPVWISMDSDGYTTDTVELAVACACAKNPSRDGGYIDLSENK

16、*Portunus trituberculatus* prepro-corazonin (full-length protein)

MVSRVTVVVLVASLALAACQTFQYSRGWTNGRKRSAELGGVVGVSSGRRAGVDFVAADPRRSLQQQTPAPRHSLPRDIEERLRAVEAGVSALLRAAQQNPEAAAAAVAGEQDYYAQN

17、*Portunus trituberculatus* prepro-CNMamide(full-length protein)

MVSRGQQGSGSLTWLLVGAVSLVLVARVQPYPGYVERLPSLRRYPQTQQQQGPAESEPFAGTNDDYVEDDDDELGYSKKYAYDTLMAGREPDIFPYDQAEASNKNLQQILRSFRPVVGGTYPSDRVLPWDDLHELGLKGGRSAEDQLTAPDTSLTQLSFAKHRQKRVMCHFKICNMGRRRRARHSNPLQGWLS

18、*Portunus trituberculatus* prepro-CRF-like_DH44(corticotropin-releasing

Factor)

MVFARLVAVLAVVSVAARGACGLSLEGVRPPHGASRPLSLAPNRRLGPRDSDLLPDDQALALMSDAAPRYEEAQLGELDDPHTSYRLKHLISELAEAIAAAEALDVGAYGGPLPAELSEALAAAEGLEGGDGRPLPSDVAEALAEAETAAVMGGSVPGMWENRDRYMLFTDYVSRPGAEANNNNNNRKARDSRNSRSVTNSNNNPSNNNNNNNSLTSTTTTSTGTSAGNNDKNMNHNSGMFKRAWPHSFSRRRNSGLSLSIDASMKVLREALYLEIARKKQRQQLQRAQHNKALLNTIGKRDVASQFAGAQHGTALGNDRN

19、*Portunus trituberculatus* prepro-diuretic hormone 31(full-length protein)

MHHFYIGLCLVVLVCVSGNAHPVDYESDEAFLSEKMREYLMLRKLLINALSERTPLKESPRKRSCYLNGLLSHGCDYQDLVSSTVEKNYWDSLNSPGKRRRRRREAQEEEI

20、*Portunus trituberculatus* prepro-ecdysis-triggering hormone(full-length protein)

MAWLVAATVLAAVVSIASADAGHFFAETPKHLPRIGRRGDLPPLTTLLSEEDA

RSSGAGTRSMTEALAGLDSDGDGCIGVAELLRIPAVRVALLLQNPALLTPANLATPEVDAHATEDTFASDRRPEPRLLRYLQK

21、*Portunus trituberculatus* prepro-elevenin(full-length protein)

MASTAPRTCLVLHTILLLTALASFASCGQAIDCRRFVFAPQCRGIIAKRTVSDSAIGLPDALQEQRQWSESIPLDYVVPEYSPAYQRVRSTYTRPSAASRDAAVGAQFSSPNAAMTEMGVRDSGDLELLSPYVKIIHRSEKLPYERK

22、*Portunus trituberculatus* pre-glycoprotein hormone-A2(full-length protein)

MVRAVVLLVACLLAYSSGYKHTWQTPGCHKVGHTRRISIPECVEFDITTNACRGYCESWSVPSAWQTLVYNPHQVVTSIGQCCNIMETEDVKVKVMCVEGPRELIFKSASTCDCFHCKKY

23、*Portunus trituberculatus* pre-glycoprotein hormone-B5(full-length protein)

MWLVQEKADPRHQVSRLEATIDGPHSCQHSPLSLAQHTPHTNTQLSTASAKIEQEEQAMTGRGGDLAGVSGRRGAGGVCGRLIAVLAAALALLLCLLPPATAIDPQSTLECHRRQYSYKVHKTDDNGRMCWDVVNVMSCWGRCDSNEIADWKFPYKRSHHPVCIHDKTQLTEVTLRNCDEGVAPGTELYSYHEATRCACAVCKSSQASCEGLRYRGARRAPRAQVPRG

24、*Portunus trituberculatus* prepro-GSEFLamide (full-length protein)

MVQGTPCLTKCIVVLSCMSCVLSAALQNTQPDESVSYVEKRAARDPMLRYLLVAMAQPGPRYAAPQILSRGVRRIGSEFLGKRSVAVSGPEKTCEPDPCVTEEGQDHADDLKKEQMSFTGQYNEQNDAGDLHDAPDKRALGDLSRSRLARYFSLLLNKKMGSEFLGKRAMGSEFLGKRAVGSEFLGKRAMGSEFLGKRAMGSEFLGKRAMGSEFLGKRAMGSEFLGKRAMGSEFLGKRAMGSEFLGKRAMGSEFLGKRAMGSEFLGKRAMGSEFLGKRAMGSEFLGKRAMGSEFLGKRAMGSEFLGKRAMGSEFLGKRAMGSEFLGKRDLWMQGPERRPEEQAEEEEEEEEREEVLSA

25、*Portunus trituberculatus* prepro-HIGSLYRamide(full-length protein)

MQGLYEDKRHIGSLYRGKKDEDSLYHVLSEDKRHIGSLYRGKKDEDNMYQSLSEDKRHIGSLYRGKKDEDNAQGLSEDKRHIGSLYRGKKDEANFAEDKRHIGSLYRGKKDEDNSQNLVEDKRHIGSLLRGKKDEDTAQGLSEDKRHIGSLYRGKKDEDNLAEDKRHIGSLYRGKKDEDNSKNILEDKRHIGSLYRGKKDEDNSQNLSEDKRHIGSLYRGKKDEDNTNSLSEDKRHIGSLYRGKKDEDNANSFAEDKRHIGSLYRGKKDDDSSSPSEDKRHIGSLYRGKKDEDNTNSLSEDKRHIGSLYRGKKDEDNTNSLSEDKRHIGSLYRGKKDEDNANSLSEDKRHIGSLYRGKKDEDNAHDLSEDKRHIGSLYRGKKDEDNANSLSEDKRHLGSLYRGKKDGQAFPTDLIQDEDDIERGLHDLPDKRYFASLLRNRQSALAPWNPPDKRHIGSFFQNRQVPFGMQDSAEKRHLGSLLQHRPSAFAPHDFTADKRHIGSFFQNRQAPFGTQDLSADKRHIGSFFQNRQSPLSLQDSAEKRHIGSFMQDRGPPFAPDQGSGLRRRKRYIGALARTNNMPSPYSRARQNRRDAPDPSRRRAHLLQRLLNMERQRDLLRQRQRQALVTRTQNAAMMLKYLEHAALDDEDVDLDLQPARDVGVDLELDEEPEPRPDLDHLQYTMAAAPLEDNALTYHSATKRFLGSLARSGWFPRRYSGAYSFDLQKRDLASQLPLDDEEEDEEEEEEEDKPFFTFSY

26、*Portunus trituberculatus* prepro-insulin-like peptide(full-length protein)

MKVVVLLLVVVTAMQTGRVRGSPRTLPEGGLVKQGERRLCGWRLANELNRVCKGVYNVPTVSTNALFYLKGRGGKRVDLWPVGGREQQFPSRTHAPADDLRASHLSEPHLFQRPADRPTGERQRLPLLTGAEASQVVVGRSPRVKRGLSAECCRKACSVSELAGYCY

27、*Portunus trituberculatus* prepro-kinin peptide

MASSLIPLLPSVMGHARVRRSINEQPHTQVDSYRHGSRRPRSLPLGGKRSGMEQGGFDDVLHAWATEGHGMAWPASVKMNVDTKRKAFSAWAGKRSGDSKHGRFSAWAGKREAFGPWKEKRSEEDEKRQAFSAWAGKRSEDEKRQAFSSWAGKREAFNAWAGKRSSNDNDKRQGFSAWAGKRSNNDNEKRKPFSAWAGKRSDGNNNKRQALNVWAGKRSNSDYDKRQAFSAWAGKRSNNDNKRQGFSAWAGKRNDNEDKRQGFSAWAGKRKFNAWAGKRSDHDYYKEQEEEEEEEKSKDQLSSLLQQHHQQEHQQASSLMHHSPDSLTHWDANWDR

28、*Portunus trituberculatus* prepro-myosuppressin (full-length protein)

MVFRLQPWCSLLLVGVVVVLGVCAGVGETIPPPICFNQKLVLTPYARRLCAALNDISKFSRAMEDYLDAQAIKNSMGVNEPEVKRQDLDHVFLRFGRAQQ

29、*Portunus trituberculatus* pre-neuroparsin(full-length protein)

MTPSARPATLILASCLLLLLLLLPRGSAAPRCTTYDQPAPKNCKYGTALDWCSNGVCAKGPGETCGGYRRQDGICGEGTYCECGHCRGCSPFDASCHDAQFC

30、*Portunus trituberculatus* pre-neuroparsin3(full-length protein)

MDNLRKAPYLIMIVSLLFFPSNIFSTPLCSSDNEVQPSECPHGTVTNRCGNTVCAKGLREPCMVYRWERDLCGGGTFCGCGFCMGCNNNLQCWDCESSAAGR

31、*Portunus trituberculatus* pre-neuroparsin2(full-length protein)

MSGGASLLLWMLIVMMVGMSSRCRALSCGPCNLNSCPDVSRCTHGTVRSVCDCHVCRKGPGESCGGPWGTGGHCKEGLKCLPNFNVAHLPKELTKHLMGTCKVVPEAPSC

32、*Portunus trituberculatus* prepro-neuropeptide F I (full-length protein)

MRGTLTVVAVVVVSVAAAWAAQLPNRQEGGALDAFQALHEAALAGTLSSGEVPYPSRPNVFKSKGELRRYLDALNAYFAIAGRPRFGKRGEQVRQPEELYDY

33、*Portunus trituberculatus* prepro-neuropeptide F II (full-length protein)

MCRQLLTALVVGVVIMGALEMGGAEAKPDPTQLAQMADVIKYLHELDKYYSPMSRPSPRSAPGPASQIQALENTLKFLQLQELGKKYSHVTRPRFGKRSEMVLPPGDALVSVRDQQMEASERLLETLARRR

34、*Portunus trituberculatus* prepro-short neuropeptide F (full-length protein)

MGVNGVKCWVALVCCCLLLCQLTTAAPADYDTLNDMYDLLAVHEVERRAPPSMRLRFGKRDMGWQVSQRSMPTLRLRFGKRNVDEADPILDHHDLIRKDARTPALRLRFGKRGASFGEEDMASQEQ

35、*Portunus trituberculatus* prepro-vasopressin (full-length protein)

MQSGVTVTVVVTLLVGSAAACFITNCPPGGKRSGGLMSTLGRARTCASCGPGLLGRCIGPDICCGARIGCFLGSRETRLCRTENMVPITCYNSDLKPCGRMQEGRCAAPGICCTENKCETNDDCVAEDTPAEEVAETQRSGRPRLDLLTAARDRWEEQ

36、*Portunus trituberculatus* prepro-orcokinin peptide(full-length protein)

MTRDVFCTALLLALCVMASEGAIKDAPTHANNHPDAGYPSDGSSAKRFDAFTTGFGHSKRNFDEIDRSSFGFAKRNFDEIDRSSFGFNKRNFDEIDRSGFGFAKRNFDEIDRSGFGFAKRNFDEIDRSSFGFNKRNFDEIDRSSFGFVKRMLTPRDIANLYKRNFDEIDRSGFGFVRRNAE

37、*Portunus trituberculatus* prepro-pigment dispersing hormone I (full-length protein)

MRSGVFVAVLVLVALAALLTKGQELHVPEREAVAALAARILKVVHAPQDGAAGLPHKRNSELINSLLGISALMNEAGRR

38、*Portunus trituberculatus* prepro-pigment dispersing hormone Ⅱ (full-length protein)

MRSAVVVAVLVVVALAALLTQGQELKYQEREMVAELAQQIYRVAQAPWAA

AVGPHKRNSELINSILGLPKVMNDAGRR

39、*Portunus trituberculatus* prepro-proctolin (full-length protein)

MARAGFLVVLVALVVLAAAVCQARYLPTRADDSRLDEIRELLRELLERTAEGGVGGSSSSSGRMAYDKRFLFKRAAPVEVAGAEVVEPLMNLPQ

40、*Portunus trituberculatus* prepro-pyrokinin (full-length protein)

MLLNPTKLLVAAPLVCLALTSTCVSLVAGAAEGAVSSPEWSARPVPPRHLGPPSPWEHAASDAVSSLLTGQINAQNVMRYQTIPERTSRPLRIMTPGVPKRLYFAPRLGKRSPSQVESLDERGRRDATSYKEDPEDAVTIPYSWWWPSVSVRRSNFSPRPGKRGEGEIDLPYDYYDPEDDEDEETEDEDEDDGVLQDKRDSTFAFSPRLGKRVQNAAFAFAPRPGKKDNFAFAPRPGKKAGTNFAFAPRPGKRTNFAFAPRPGKKSNFAFAPRPGKKTFAFSPRLGKKADFAFAPRPGKRSDSPSETGDRQTGETWWIGEGGSATVTTQPPFLPPRLE

41、*Portunus trituberculatus* prepro- red pigment concentrating hormone (full-length protein)

MVRRAGVTLLVVALVVVALVSSVSAQLNFSPGWGKRAAAASGNNGGVGEAVSGLHPSVVGAPGGVVPPGSSSSSGDSCGPIPVSAVMHIYRLIRSEAVRLVQCQDEEYLG

42、*Portunus trituberculatus* prepro-RYamide(full-length protein)

MKMPRSFSPALVLLAALIAVAASQGFYSQRYGKRGSDTRQVTERSGFYANRYGRSQGIPEIKVRSSRFVGGSRYGKRSGVPALAEVPLPVVAPESEEGEVGASLLLGDSVVCLLVDVPDIYRCLKKSASEESTN

43、*Portunus trituberculatus* prepro- SIFamide (full-length protein)

MSMQMRVVVAVAVVVVVLALLSSPVSAGYRKPPFNGSIFGKRSGADAVYEPGKSQALASVCQVALEACTLWFPGAEKK

44、*Portunus trituberculatus* prepro-sulfakinin (full-length protein)

MSPSTSSSVARRWLLWWCMAAAAAAVVGAAGEVVVGGGGSLPARPLPQRPAAALARVLAPVVRHRMEEEEEGLLEELLEEEGGLIGGAGPVEVLDAAGKREFDDYGHMRFGKRGSASDDYQDDYGHLRFGRSSHHTTHRHTTTTNNNNNNYHHRRFNALQNGGKM

45、*Portunus trituberculatus* prepro-tachykinin(-related)peptide (full-length protein)

MSRMMMWAAVVLMGVAAAAAGGAEQGEAGSDGPRPRRAPSGFLGMRGKKEAPSPSLQDAPAPPEDLLPSFYQLDVPLRGKKAPSGFLGMRGKKSEEEEEEEEEGMYARPSFNSEFDSLVKRYPSGFLGMRGKKAPSGFLGMRGKKSSIEDYLSSPVSRQALLSLLQGDAAPPMDDDYYYYYNPEGWRAGTHKRAPSGFLGMRGKKDAYPGFPQDKRTPSGFLGMRG

46、*Portunus trituberculatus* prepro-trissin (full-length protein)

MMSMQIYLAMVLAAWAVVVGTCSSSTVSCDSCGPECQTACGTKNFRACCFNFLRRRRSYSLPVRRSQGVGTAAEWKALRGALMSPAAASRGLPFAQFLEASETDPAPQPTKHHRDPSSLASVLTTLLQDSSMDEEDEDEMLELEGEGGGEGIPEASADDATLSRLVAFAFHQPPPPPRSPLNQHYPAHSPPPPPPAGDVGK

47、*Portunus trituberculatus* prepro-natalisin (full-length protein)

MASMWGLLVMALTAAFAATAQELSPGEAEGGEGQAWEGHRHARSLEVGGDASWLSPVQDTDVTQVEPRVEAGYGGTTFWVARGKKDAETFPSYYWGPNQGLWGETFQRGGMPATASSSHAPLAVKSGWESNPSLWGKRDGRGPFWATRGKRPDPFWASRGRRDSSEMPSTLLQQWAAEEPAMLEQKEERLWGGEIKREEGGPFWISRGKRPHSGSAASQLASLWAIRGRKSGADNTFWAARGKKETNVRGPFWAARGKRSGGEAGTGPYWIARGKKQDATTPTGPYWIARGKKEDDGAVFWAVRGKKDPPAWTTGRGRREDGTHSFWIARGKKNEHTASHNTEKNNDNDDDEQQEEATQKAADHYLKGFTALSDK

Supplemental Figure 4. Amino acid sequences of peptide precursor proteins deduced from *Portunus trituberculatus* transcriptomic data. In this figure, signal peptides are shown in gray, while all mono/dibasic cleavage loci are shown in yellow. The mature peptide are shown in green, with all linker/precursor related peptides shown in blue. The mature peptide of neurophysin is shown in red. The mature peptide of orcomyotropin is shown in pink.
