# Supplementary material for: Identification and characterization of expression profiles of neuropeptides and their GPCRs in the swimming crab, Portunus trituberculatus
Source: PeerJ. 2021 Sep 15;9:e12179. doi: 10.7717/peerj.12179 (PMC8449533; doi:10.7717/peerj.12179)

Full-length gels which have been cropped in the main text

**RT-PCR results in Figure 10 of the main text, red lines represent cropping lines. All gels have been run under the same experimental conditions. DL 1,000 DNA Marker has been used in the experiment. From top to bottom in the lane, the DNA marker size is 1,000bp, 700bp, 500bp, 400bp, 300bp, 200bp and 100bp.**

1.ACP


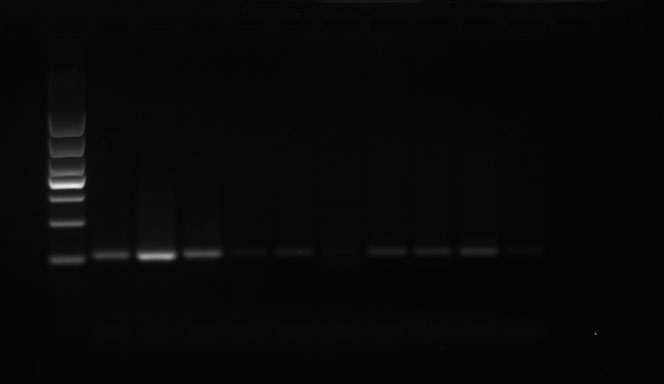


2.ALP


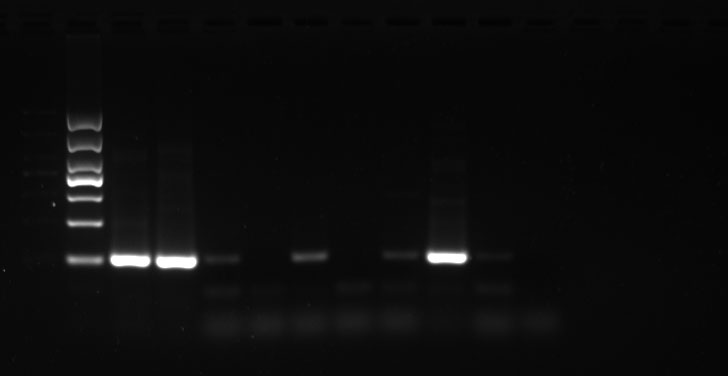


3.AST-A


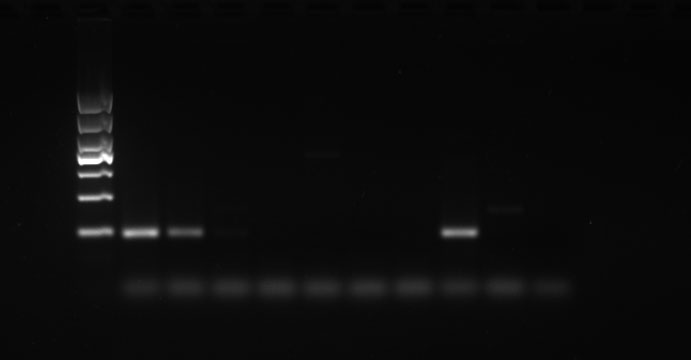


4.AST-B


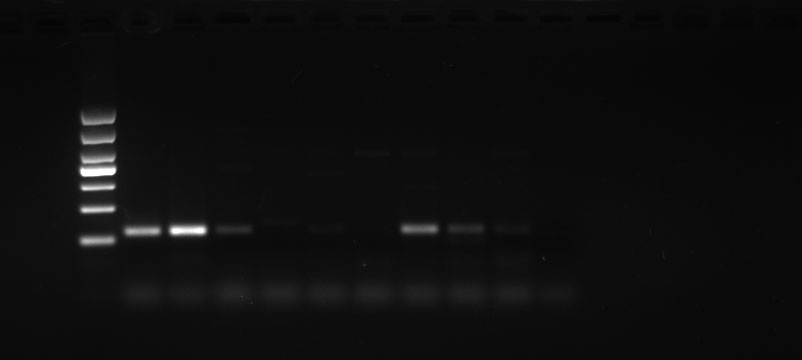


5.AST-C


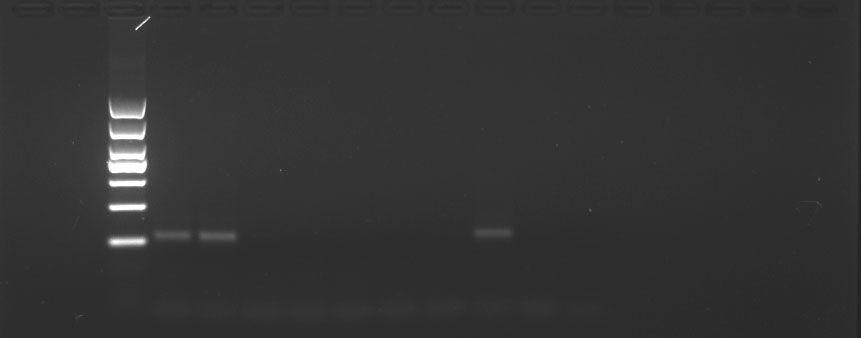


6.AST-CCC


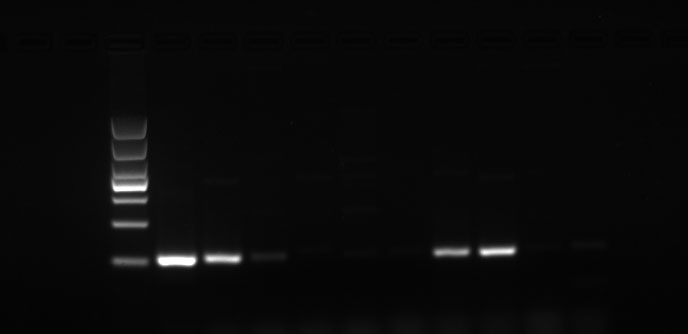


7.CCAP


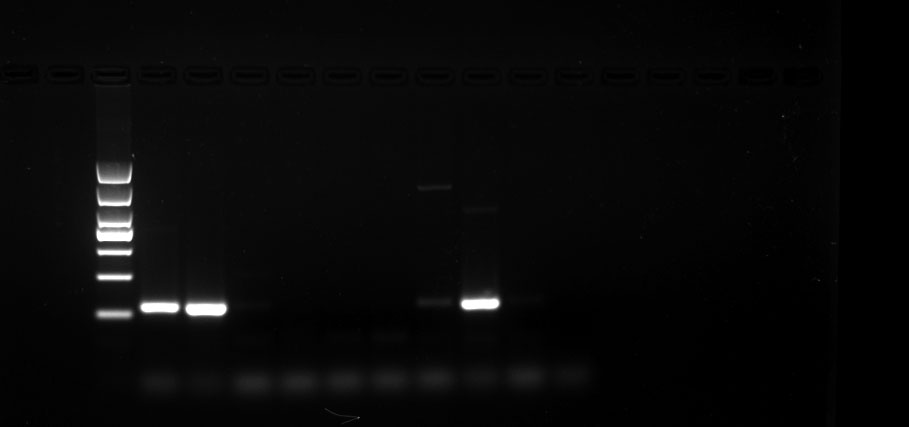


8.CHH1


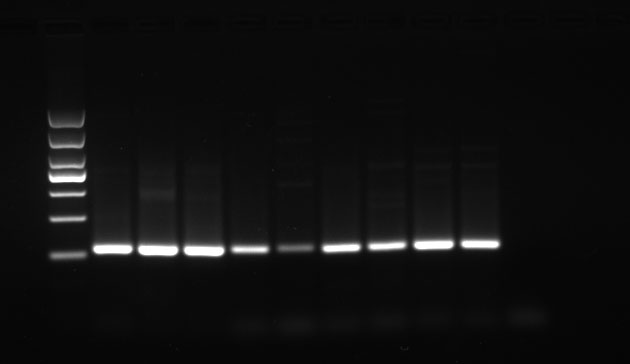


9.CHH2


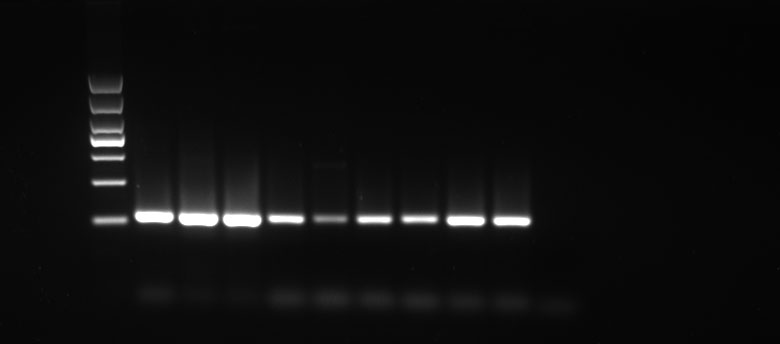


10.MIH


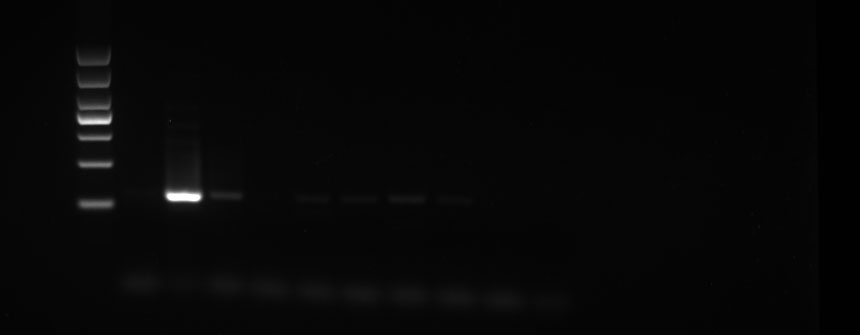


11.CFSH


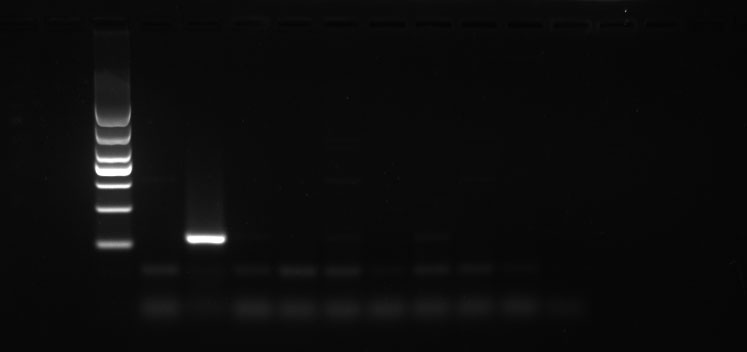


12.Corazonin


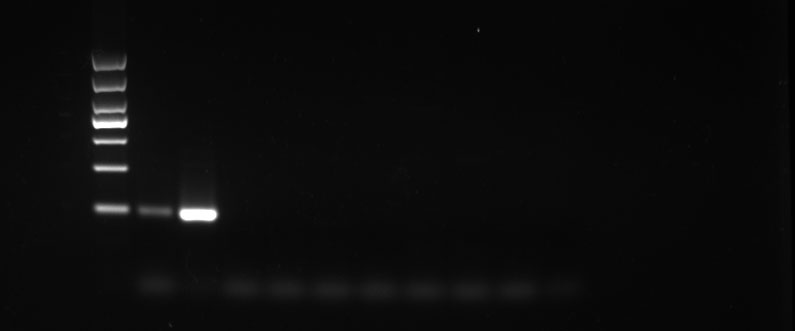


13.CNM


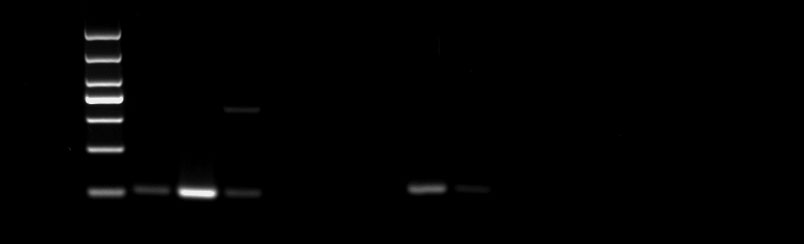


14.DH31


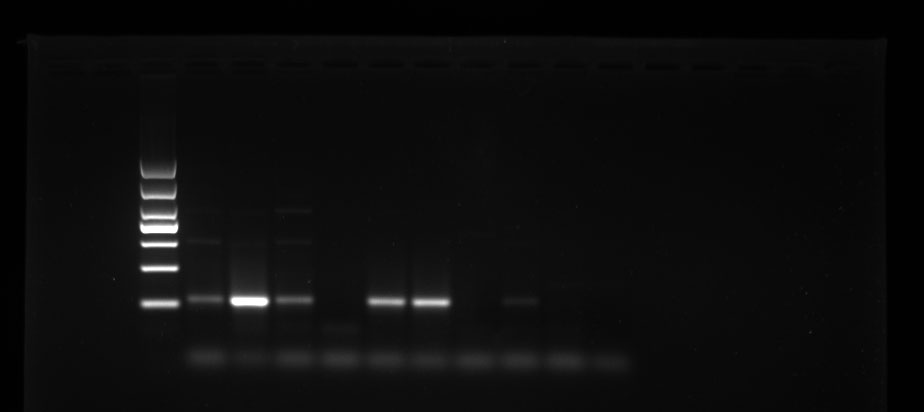


15.Elevenin


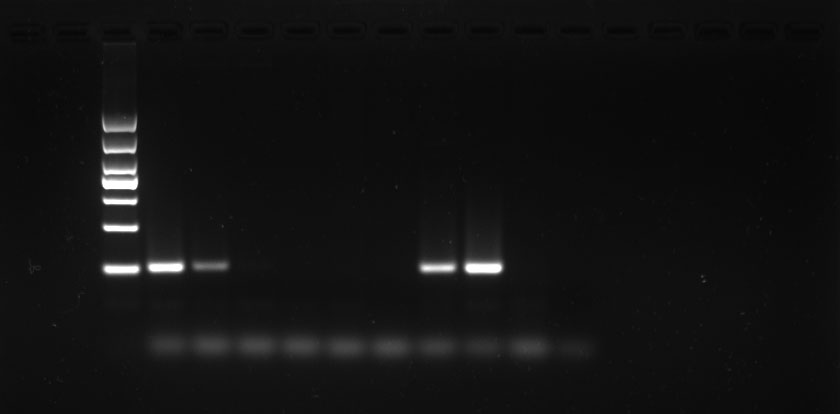


16.GPA2


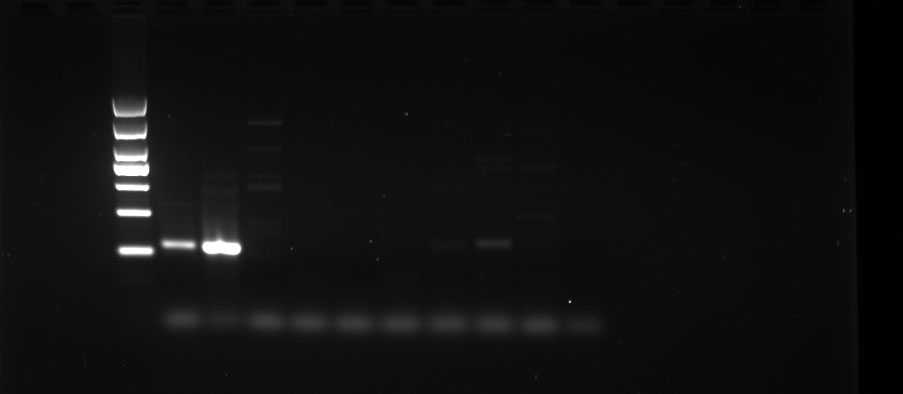


17.GPB5


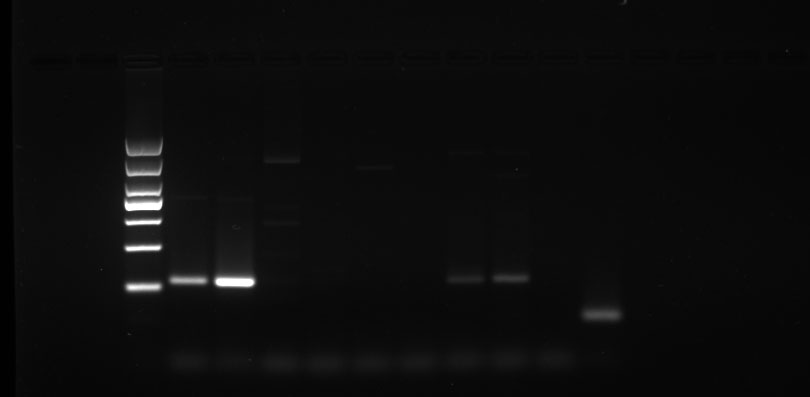


18.GSE


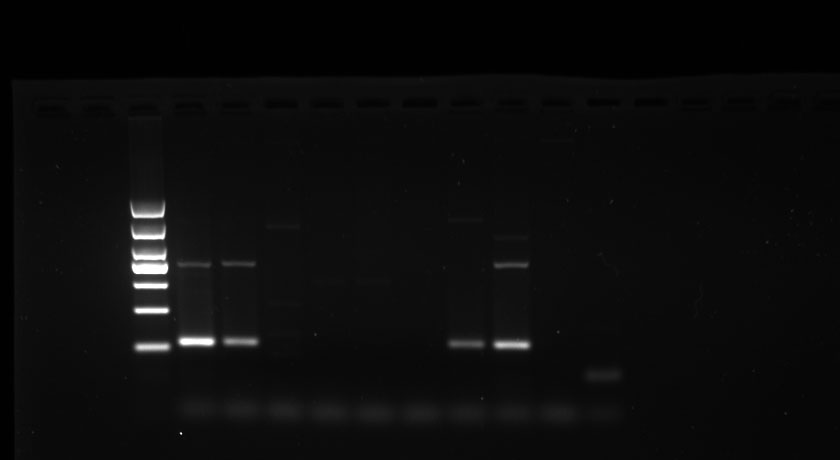


19.Kinin


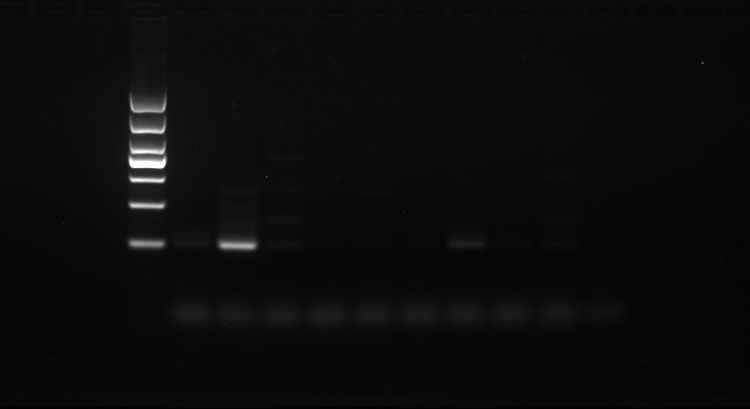


20.Myosuppressin


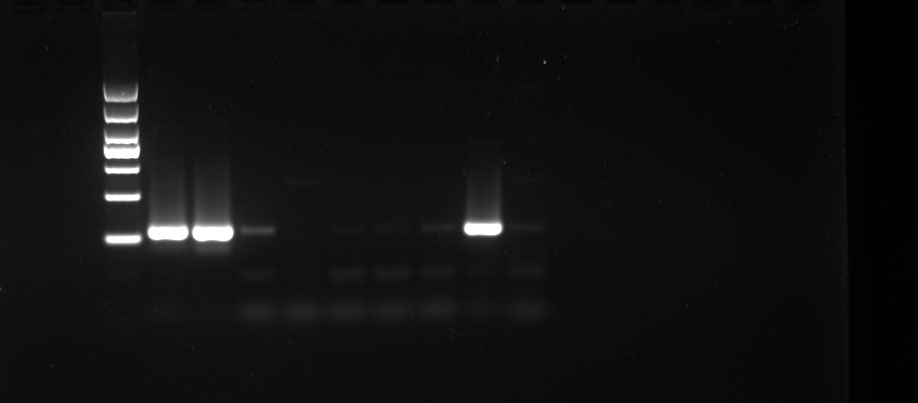


21.NP1


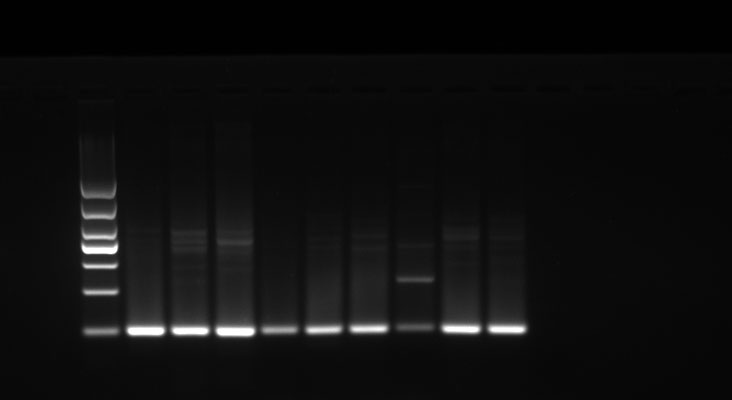


22.NP2


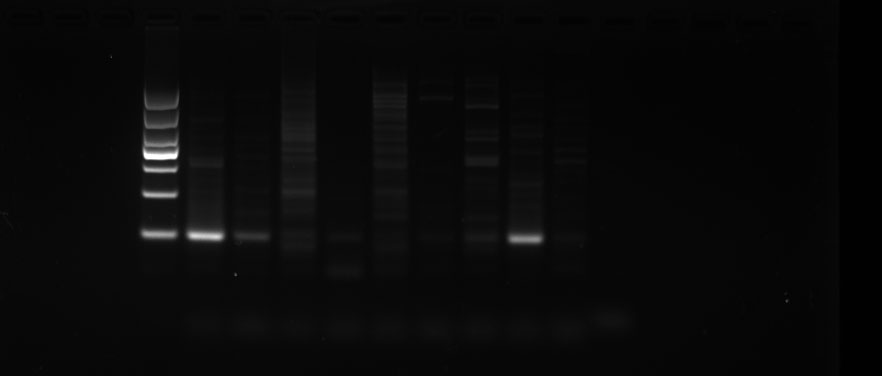


23.NP3


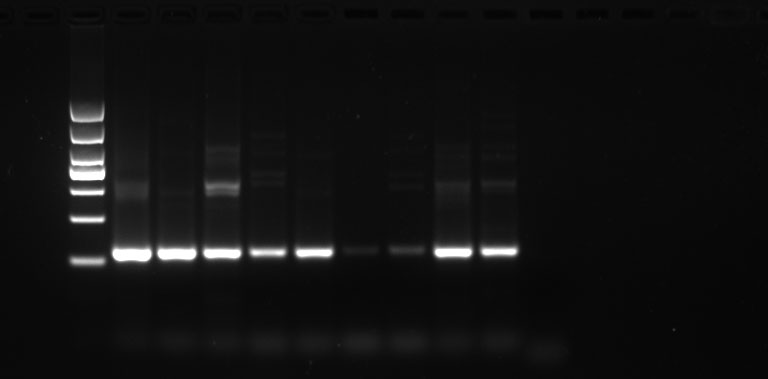


24.NPF1


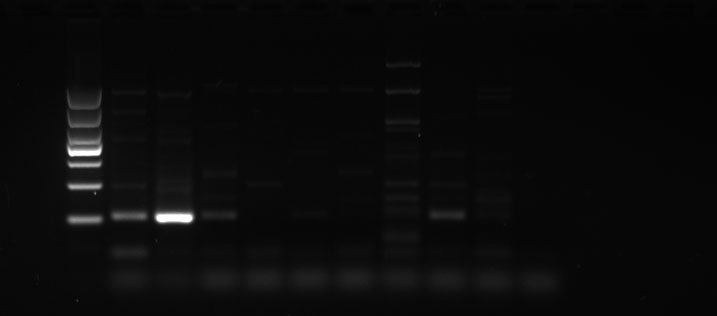


25.NPF2


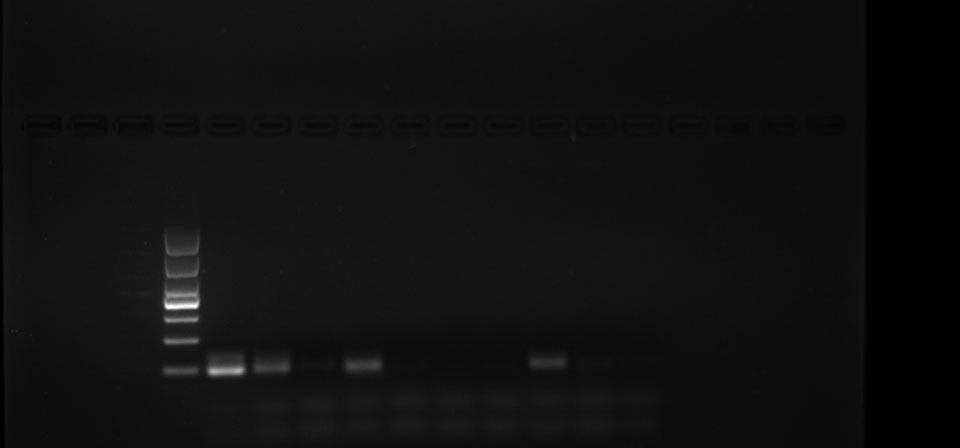


26.sNPF


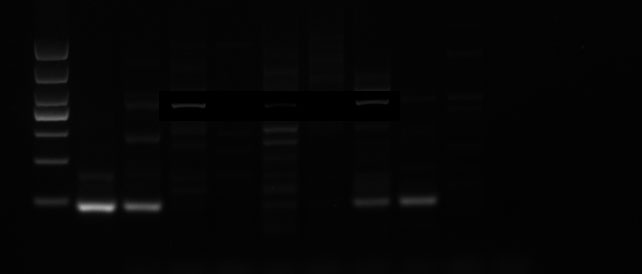


27.PDH1


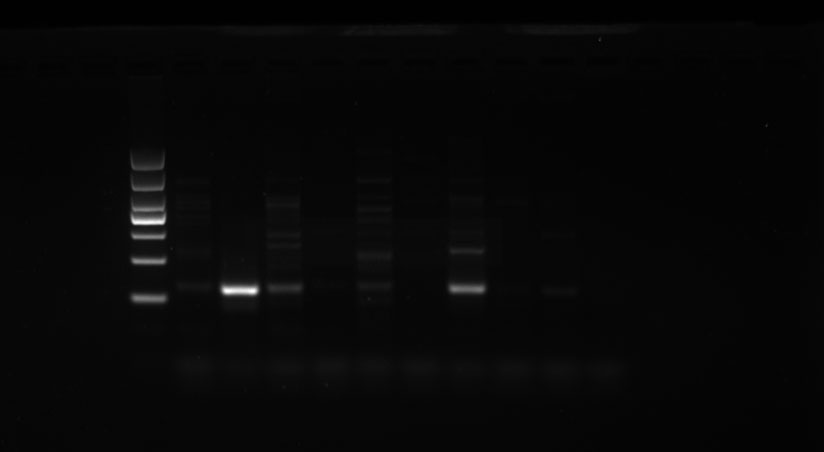


28.PDH2


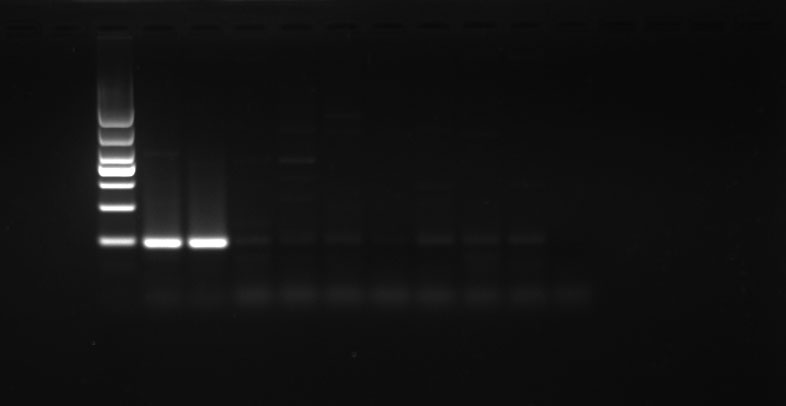


29.RYamide


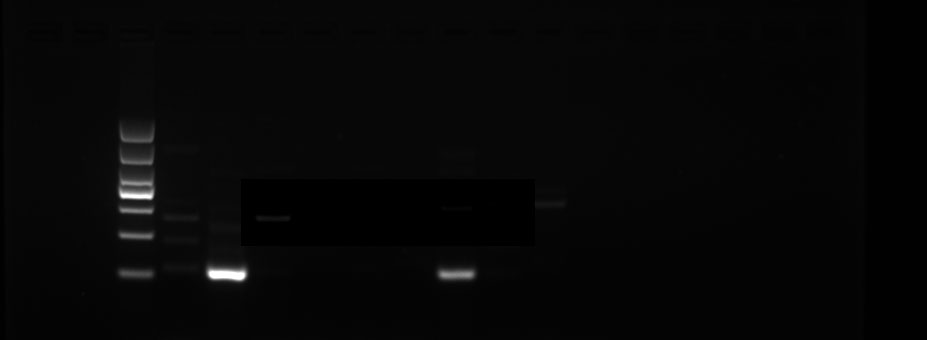


30.RPCH


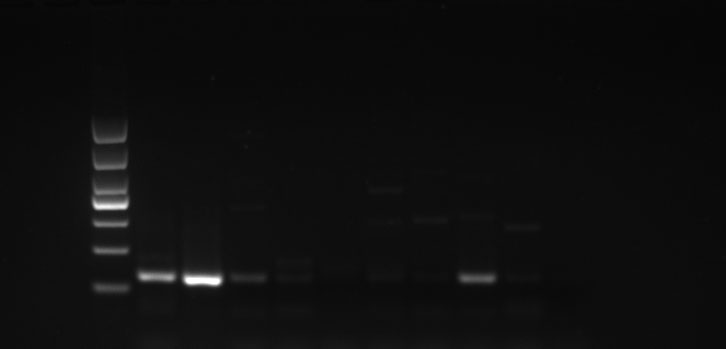


31.Tachykinin


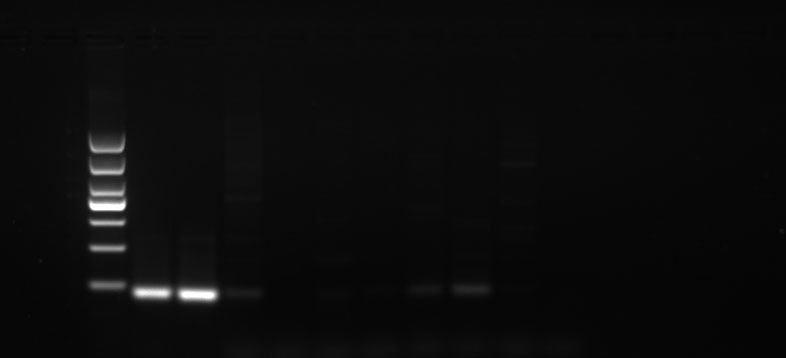


32.Trissin


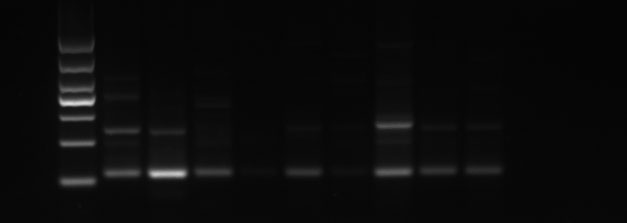


33.β-actin


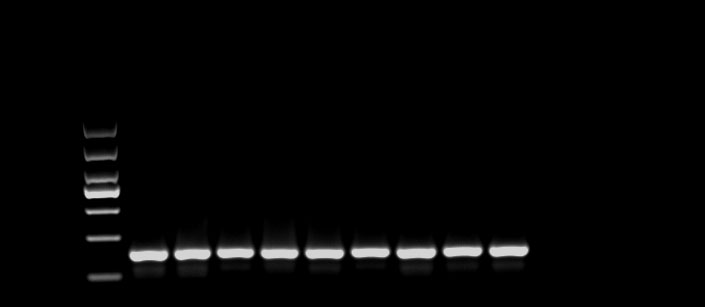

Supplement: Supplemental Information 5 — RT-PCR results in Fig. 10 of the main text, red lines represent cropping lines. All gels have been run under the same experimental conditions. [file peerj-09-12179-s005.docx]
