# Supplementary material for: Identification and characterization of expression profiles of neuropeptides and their GPCRs in the swimming crab, Portunus trituberculatus
Source: PeerJ. 2021 Sep 15;9:e12179. doi: 10.7717/peerj.12179 (PMC8449533; doi:10.7717/peerj.12179)

Full-length gels which have been cropped in the main text

**RT-PCR results in Figure 11 of the main text, red lines represent cropping lines. All gels have been run under the same experimental conditions. DL 1,000 DNA Marker has been used in the experiment. From top to bottom in the lane, the DNA marker size is 1,000bp, 700bp, 500bp, 400bp, 300bp, 200bp and 100bp.**

1.Pt-GPCR-A1


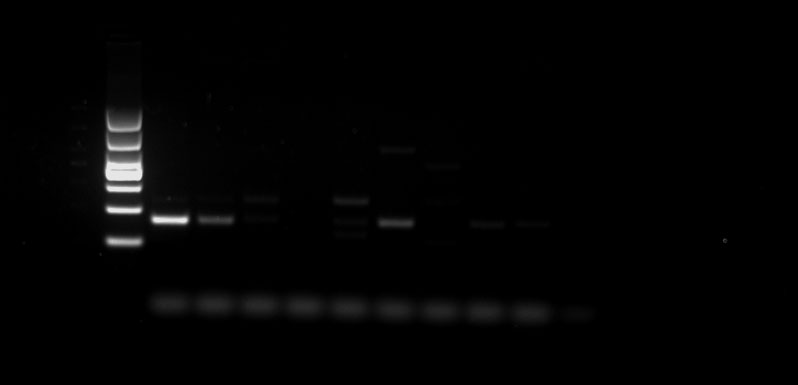


2.Pt-GPCR-A2


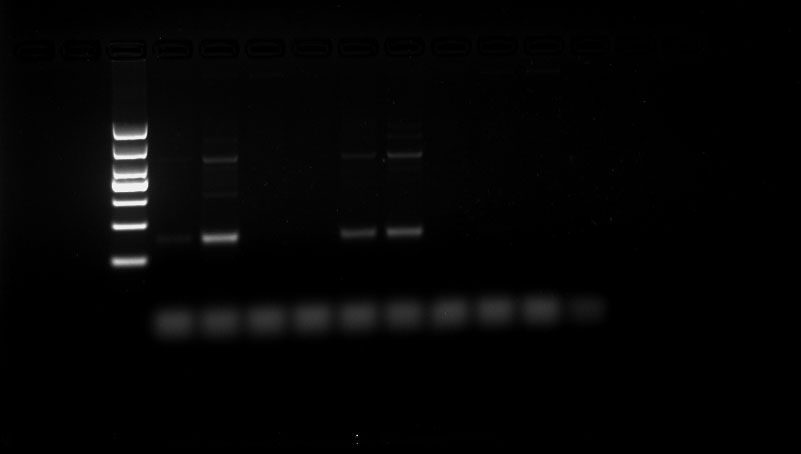


3.Pt-GPCR-A4


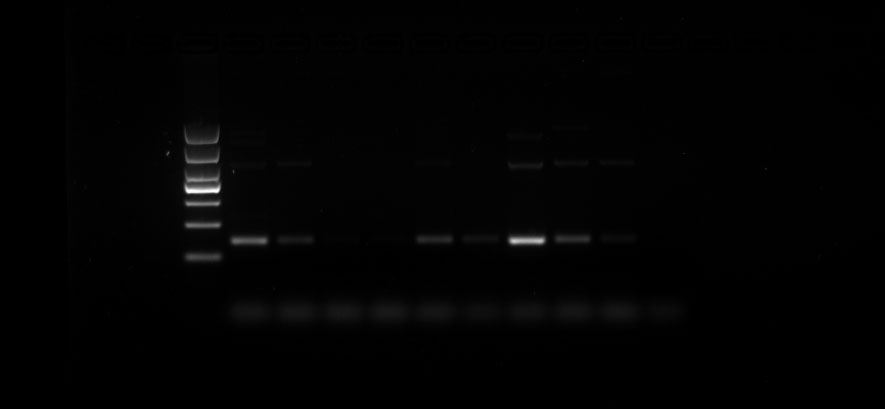


4.Pt-GPCR-A7


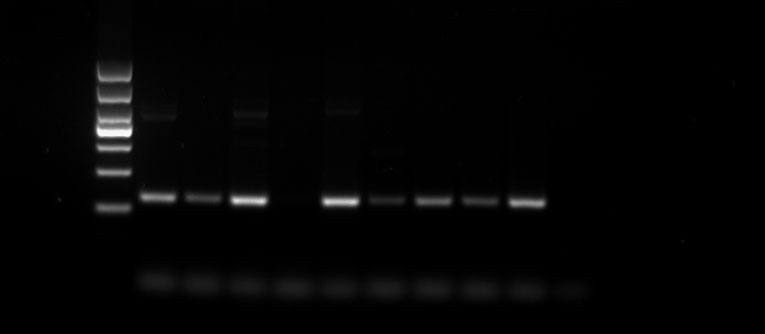


5.Pt-GPCR-A8


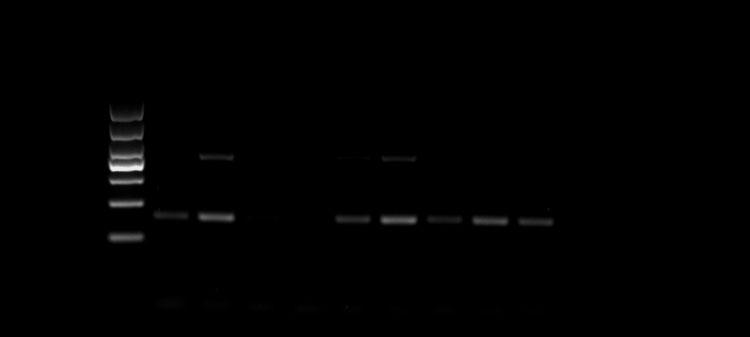


6.Pt-GPCR-A9


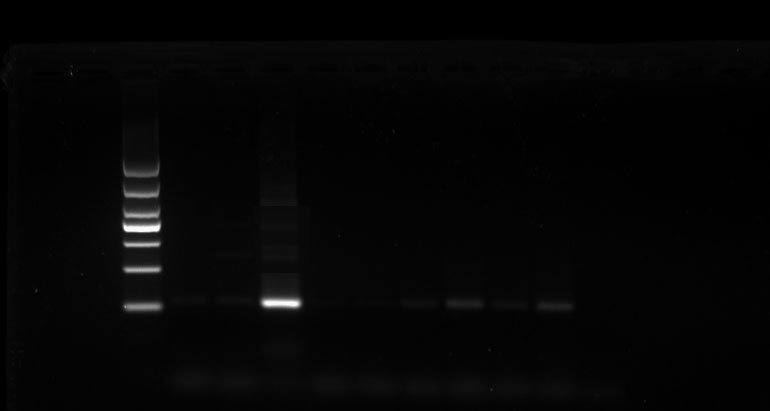


7.Pt-GPCR-A11


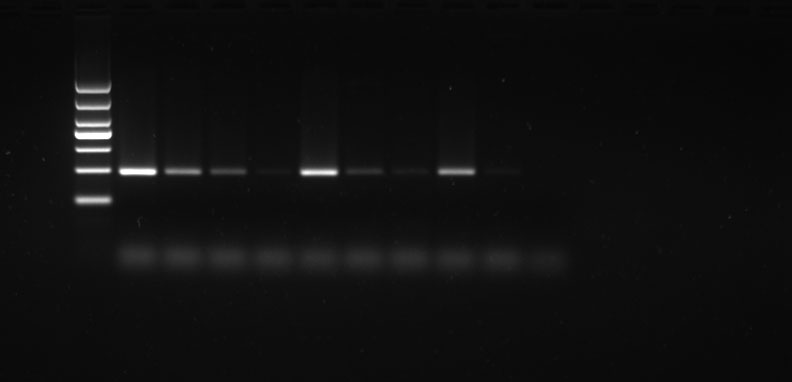


8.Pt-GPCR-A12


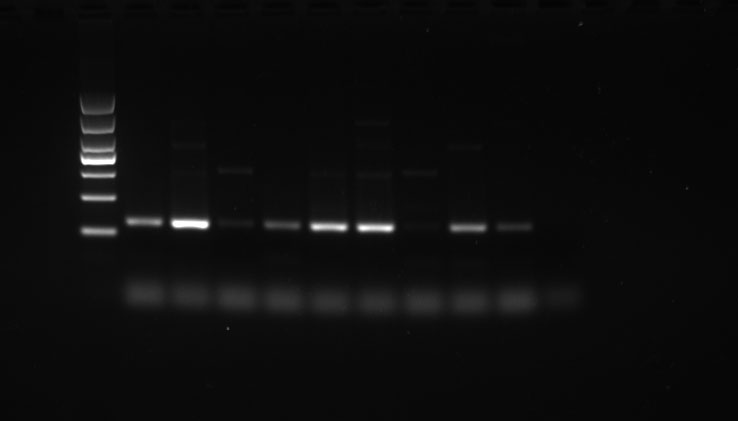


9.Pt-GPCR-A13


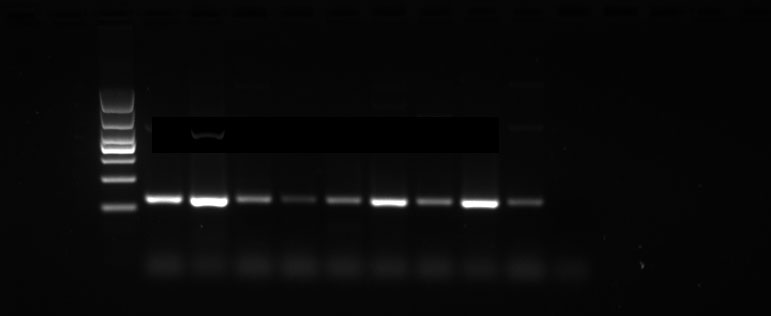


10.Pt-GPCR-A14


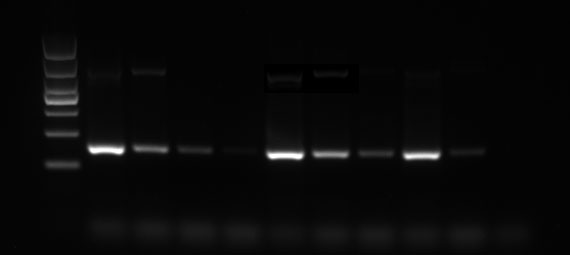


11.Pt-GPCR-A16


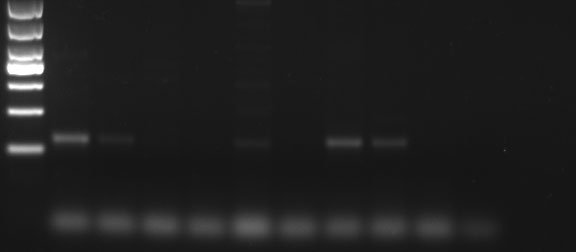


12.Pt-GPCR-A18


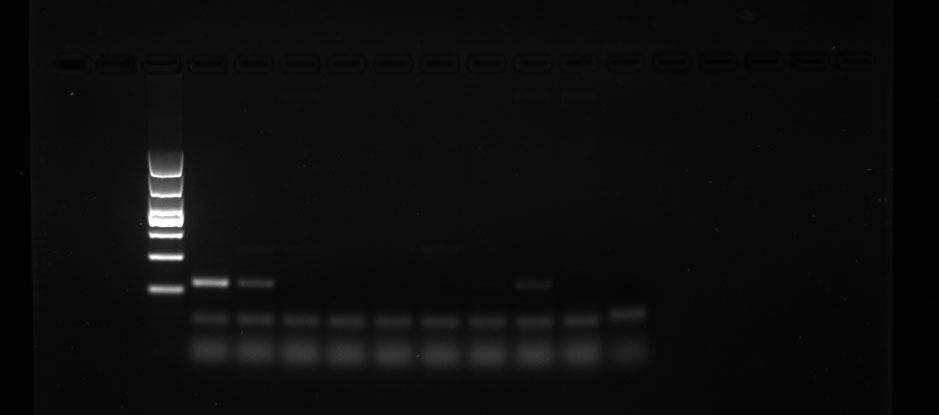


13.Pt-GPCR-A19


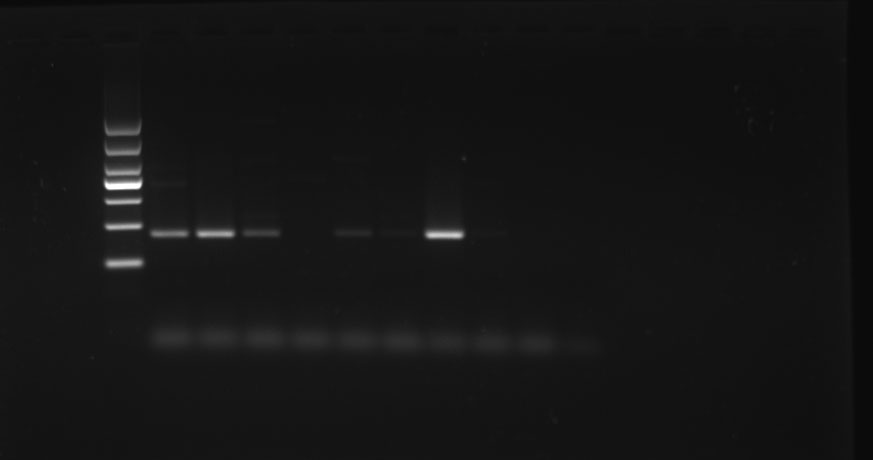


14.Pt-GPCR-A21


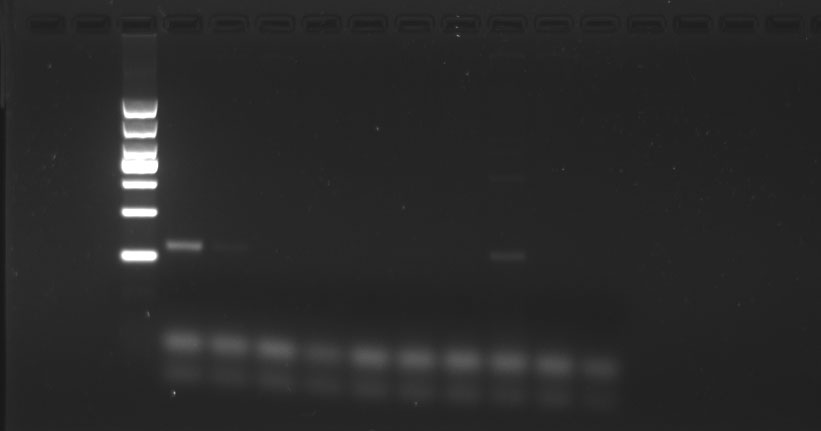


15.Pt-GPCR-A23


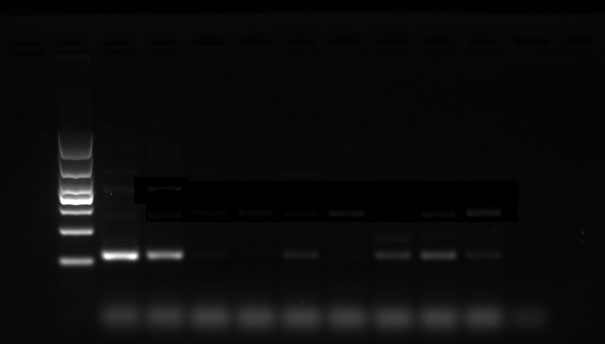


16.Pt-GPCR-A24


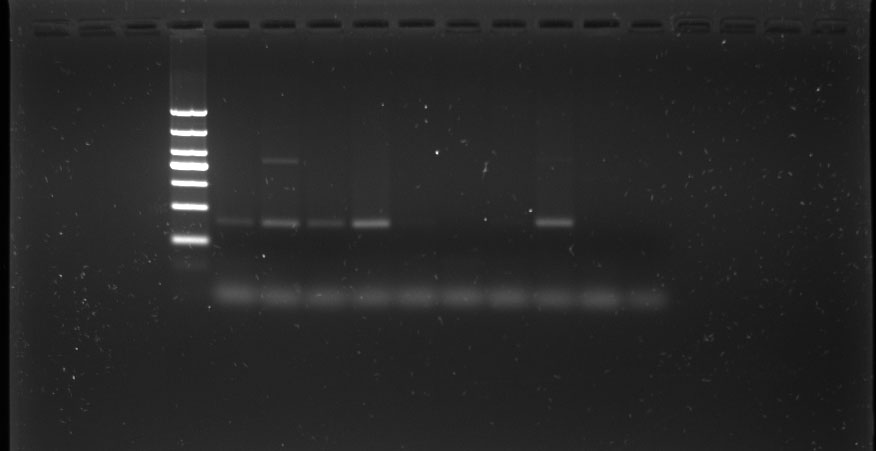


17.Pt-GPCR-A25


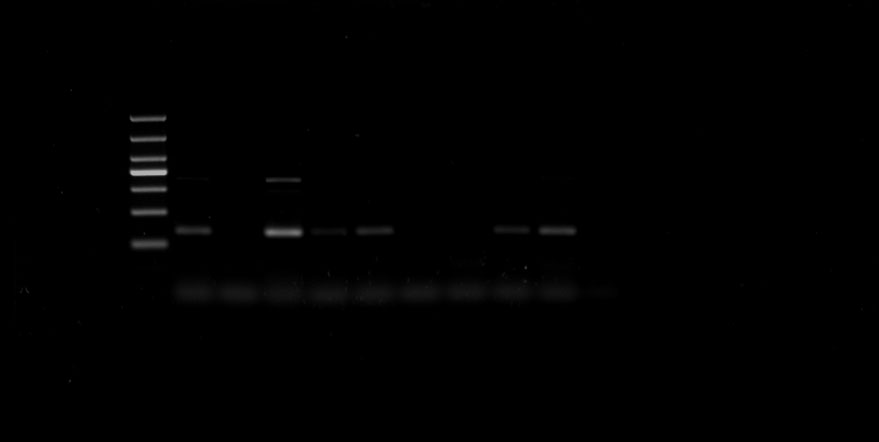


18.Pt-GPCR-A26


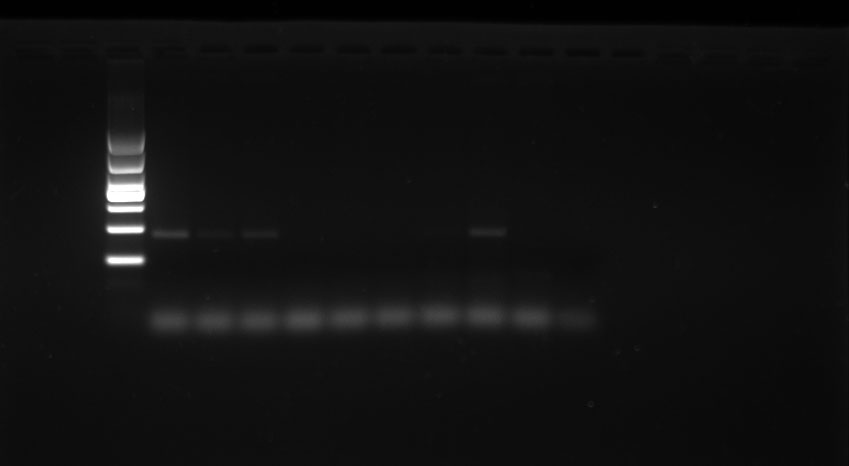


19.Pt-GPCR-A27


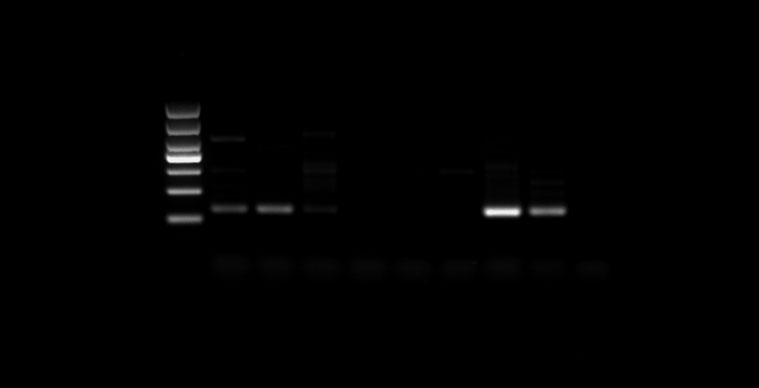


20.Pt-GPCR-A28


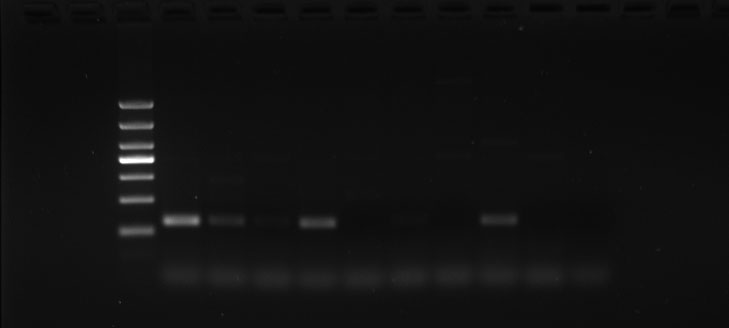


21.Pt-GPCR-A29


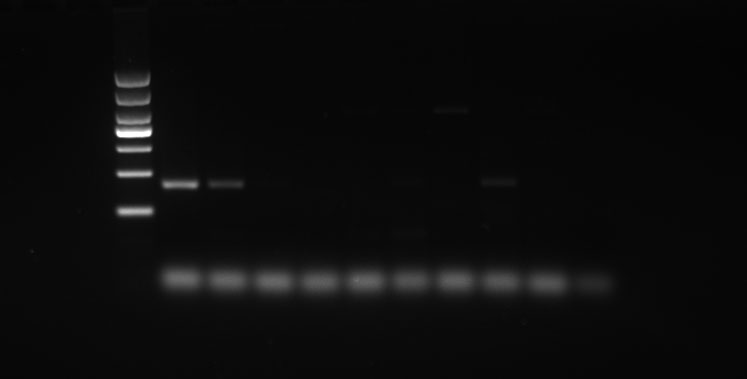


22.Pt-GPCR-A32


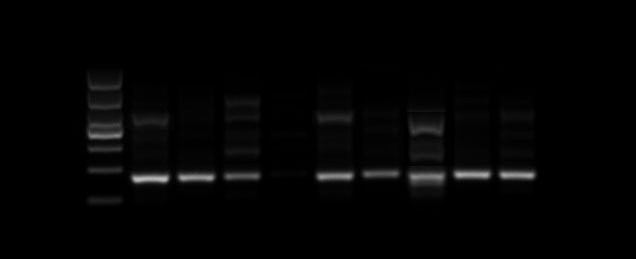


23.Pt-GPCR-A33


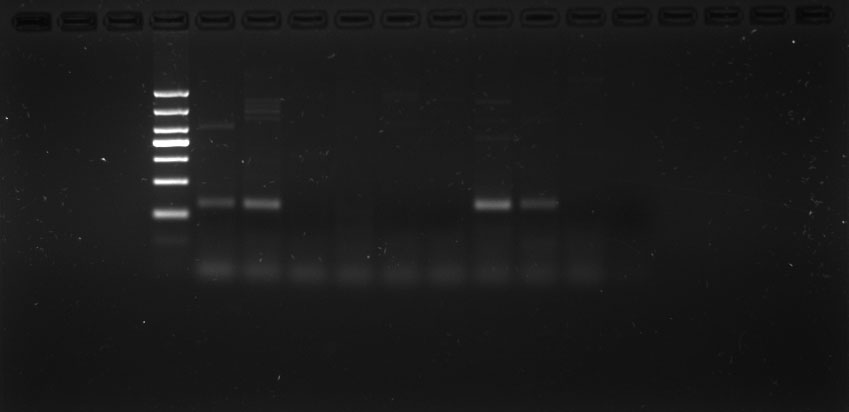


24.Pt-GPCR-A34


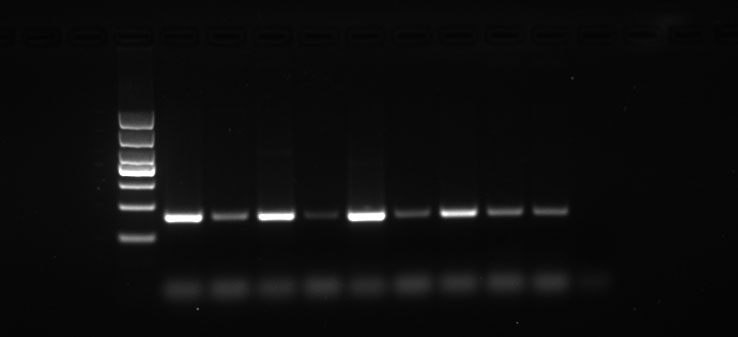


25.Pt-GPCR-A36


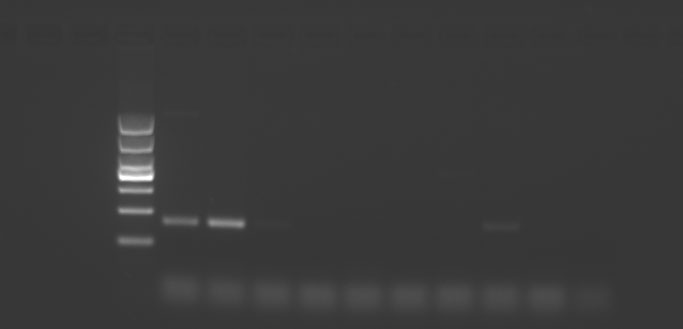


26.Pt-GPCR-A37


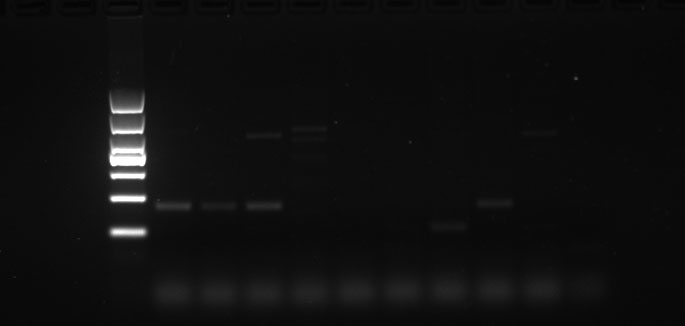


27.Pt-GPCR-A39


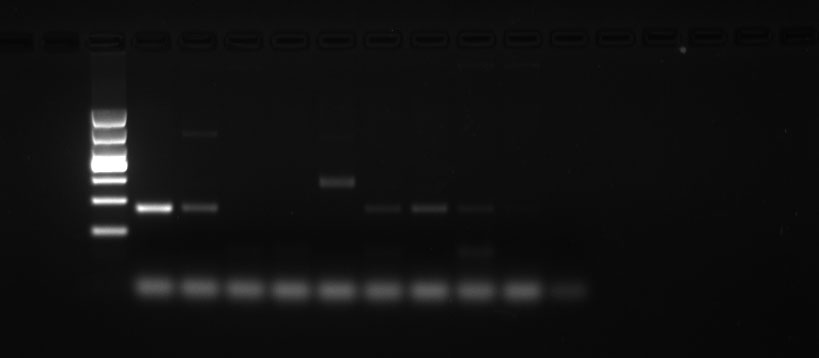


28.Pt-GPCR-B2


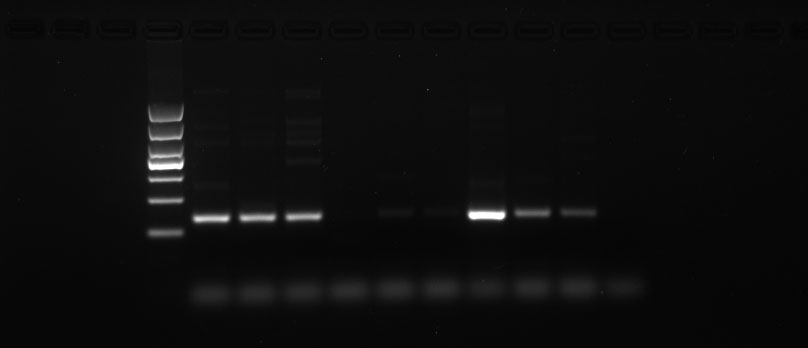


29.Pt-GPCR-B4


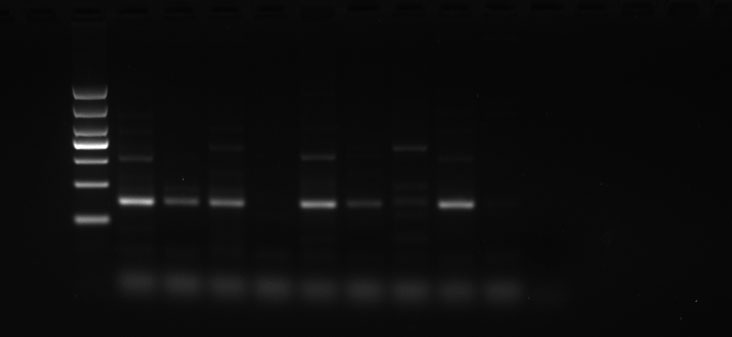


30.Pt-GPCR-B5


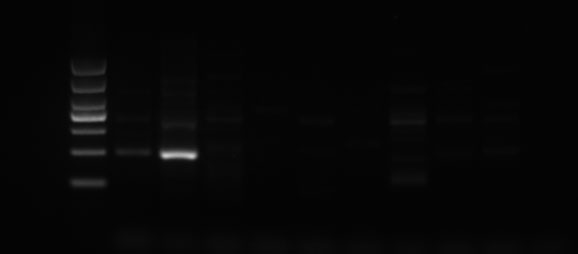


31.Pt-GPCR-B6


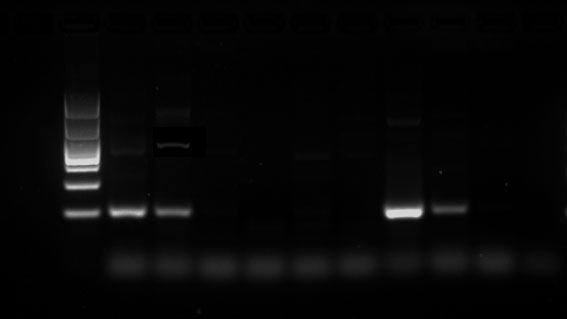


32.Pt-GPCR-B7


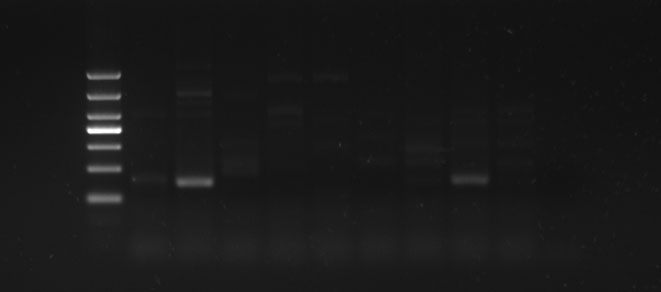


33.Pt-GPCR-B8


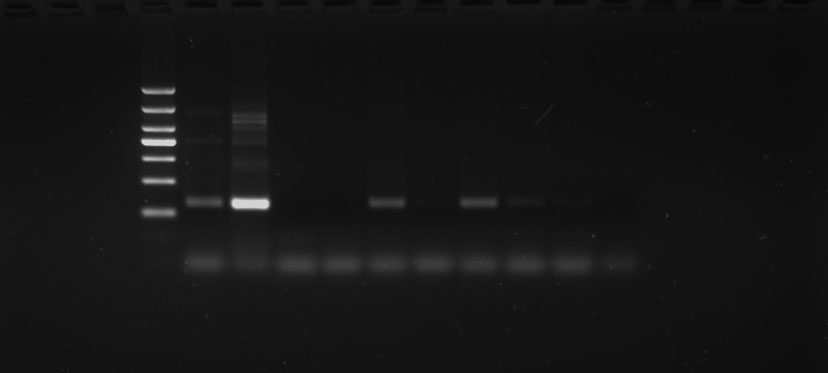


34. β-actin


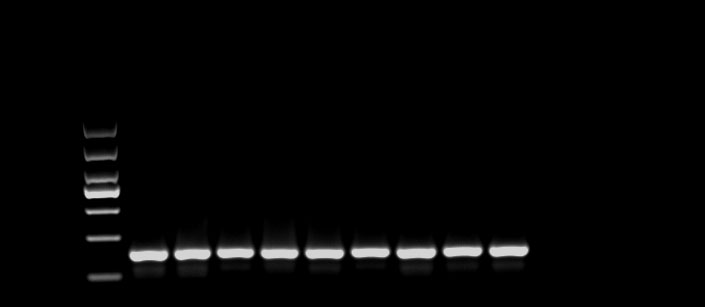

Supplement: Supplemental Information 6 — RT-PCR results in Fig. 11 of the main text, red lines represent cropping lines. All gels have been run under the same experimental conditions. [file peerj-09-12179-s006.docx]
