# Supplementary material for: Identification and characterization of expression profiles of neuropeptides and their GPCRs in the swimming crab, Portunus trituberculatus
Source: PeerJ. 2021 Sep 15;9:e12179. doi: 10.7717/peerj.12179 (PMC8449533; doi:10.7717/peerj.12179)
Supplement: Supplemental Information 7 [file peerj-09-12179-s007.docx]

> *Portunus trituberculatus* putative Eclosion hormone nucleotide sequence

GCTAGAGAAGAGGTAAGAAAAGAGAGAGTGGTGAGGAATGAGAAAGGGAAGGAAGGGTTGAGGGTTGAGAACATAAGGGAAAGGGAGGGGAGGTTTGAGAATAAGGGTGTGAAGGGAAGGGGGGAGAGGGAATTGGGCACTAGTCCACTGACCAGCTTCCTCACCGCCGGCCCAGAATGGTTGGCTCCAGAAAGGTCGTCGTGTCGGCCCTGCTGGTGCTGAGCGTGGCGCTGGTGCTGGCGGTGCTGCTGTTGCCGCCGTCAGCCTCCGCAGCCGTCGCCGCCAACAGGAAGGTCTCCATCTGCATCAAGAACTGCGGCCAGTGTAAGAAGATGTACACTGACTACTTCAACGGCGGACTCTGCGGGGACTTCTGCCTCCAGACTGAGGGCCGCTTCATCCCGGACTGCAACCGCCCGGACATCCTCATCCCGTTCTTCCTCCAGCGACTAGAGTGAAGGAGGCGCGGCGACGCAAGATGATGCCGTGGTGTTGTGGCGCCTGACGCACCGCCCCTCGAGATGCACACACGTCCCCACACCGTGTTGGTCGTCGGCAGCGGCAGGAGAAGGGACATGACGACGGTGCTGCCCTGTGGCTGACGCCCTCTTACCCTATCTCCGAGTCACGTGCACGCAGGTCAAGTCGTCACGGTGAAAGCTGCCGCGTGCCTCGCCCCGCTGTCCGCTGAACGCTCACAAAACAGCGGTGATGGAGAGCAGCTGTACCAGAAATCAGACTGCAGCTCCACCATCCACATCTATCCACACCCATCCACCCACCCTGGGGTATCCGTGCCCGGGGACTAACAGGAAACGGGGATGCTTCAAGTGACAGCCTGGTGTTTCAAACTGGCACTCTGTCACGGCGGAATCCAACACACCCCCTCCCTTCCTCGCAGACCCCACACGGAGTCCCCACACCACACTCTCCCTAAGTTTAGTTTCTTTAGTCCCATGAGAGTCACTGCTGAGATGTACATGTATATTTTTCTACAACTCTTTGCATTTTCAGTAAATATACCCAAGTGAAGGCTGTGTTTGTGTCATGCCCTGGACACCTGCGAAACCATGAACACACACACATGATAGAGTG

> *Portunus trituberculatus* prepro- Eclosion hormone

MVGSRKVVVSALLVLSVALVLAVLLLPPSASAAVAANRKVSICIKNCGQCKKMYTDYFNGGLCGDFCLQTEGRFIPDCNRPDILIPFFLQRLE

Supplemental Figure 7. Eclosion hormone transcript and amino acid sequences of peptide precursor proteins deduced from *Portunus trituberculatus* testis transcriptomic data. In this figure, signal peptides are shown in gray. The mature peptide are shown in green.
